# Exploring treatment preferences facilitated recruitment to randomized controlled trials

**DOI:** 10.1016/j.jclinepi.2010.12.017

**Published:** 2011-10

**Authors:** Nicola Mills, Jenny L. Donovan, Julia Wade, Freddie C. Hamdy, David E. Neal, J. Athene Lane

**Affiliations:** a School of Social and Community Medicine, University of Bristol, Canynge Hall, 39 Whatley Road, Bristol BS8 2PS, UK; b Nuffield Department of Surgery, University of Oxford, John Radcliffe Hospital, Oxford OX3 9DU, UK; c Department of Oncology, University of Cambridge, Addenbrooke’s Hospital, Hills Road, Cambridge CB2 0QQ, UK

**Keywords:** Treatment preferences, Prostate cancer, ProtecT study, Qualitative research methods, Randomized controlled trial, Recruitment to RCTs

## Abstract

**Objective:**

To explore how patients' treatment preferences were expressed and justified during recruitment to a randomized controlled trial (RCT) and how they influenced participation and treatment decisions.

**Study Design and Setting:**

Qualitative analysis of audio recordings of recruitment appointments with 93 participants aged 51–70 years in a UK multicenter RCT of localized prostate cancer treatments.

**Results:**

Treatment preferences at recruitment were more complex and dynamic than previously assumed. Most participants expressed views about treatments early in appointments, ranging on a continuum from hesitant to well-formed opinions. As recruiters elicited men’s views and provided detailed evidence-based treatment and study information, some opted for their preference, but many became uncertain and open to RCT recruitment, often accepting a different treatment from their original “preference.” Discussion of treatment preferences did not act as the expected barrier to recruitment but actively enabled many to express their concerns and reach an informed decision that often included RCT participation.

**Conclusion:**

Exploring treatment preferences and providing evidence-based information can improve levels of informed decision making and facilitate RCT participation. Treatment preferences should be reconceptualized from a barrier to recruitment to an integral part of the information exchange necessary for informed decision making about treatments and RCT participation.

## 1 Introduction

**What is new?**

• Patients' treatment preferences expressed during randomized controlled trial (RCT) recruitment were found to be more complex and dynamic than previously assumed in the literature, ranging on a continuum from hesitant views to strong intentions to receive a particular treatment.
• Exploration of treatment preferences by recruitment staff facilitated recruitment by helping potential trial participants to express their concerns, focus views, and reach an informed decision about RCT participation or choice of treatment.
• Patients' treatment preferences should be reconceptualized from a “barrier” to trial recruitment to an integral part of the information exchange necessary for informed decision making about treatments and trial participation.
• Future research should focus on developing strategies to support trial recruiters in carefully eliciting and exploring treatment preferences so that they can provide targeted information to those who need it most.

Randomized controlled trials (RCTs) are increasing in number and complexity to tackle key evaluative health care questions, but overcoming recruitment difficulties and increasing participation rates are still a challenge [1]. Low rates of recruitment may threaten the external validity of RCTs [2], lead to the need for considerable further resources, or cause trials to end prematurely, leaving important research questions unanswered. Recruitment to RCTs should only occur when there is “equipoise”—uncertainty over the most effective treatment [3]—and when potential recruits have been given sufficient information to make an informed choice about participation [4]. Patients’ treatment preferences have been identified as a barrier to trial recruitment and one of the major reasons for low participation levels [5–7]. A recent systematic review showed that substantial numbers of potential recruits refused randomization because of treatment preferences, particularly those who were employed and well educated [7].

Although the impact of patients’ treatment preferences on RCT recruitment is thought to be considerable, research to understand these preferences is meager and lacks theoretical insight [8]. The vast majority of studies that assessed the impact of treatment preferences on randomized trials identified through recent systematic reviews have assumed that preferences were easily defined and measured [7,9]. “Simple preferences” have been elicited “whereby the participants indicated which treatment they preferred” [9,p. 5] using “very simple measures” [10,p. iii] such as single-item scales, with little consideration of validity, reliability, or sensitivity [11], or what was being measured. However, a small but significant body of literature emphasized the complexity of treatment preferences, revealing them as multifaceted psychological phenomena that could change over time and required rigorous assessment in trials [11–13]. Moreover, studies have shown that the way in which information about different treatments in both clinical practice and trials is presented to patients, for example, positively or negatively framed survival probabilities and verbal or numerical risks of disease recurrence, shapes their attitude toward the treatments offered [14–18]. A recent conceptual framework to understand patients' treatment preferences and their effects on decision making in RCTs further highlighted the complex nature of preferences [19]. The framework proposed the development of preferences within trials as a four-stage process relating to information, reasoning, judgment, and decision making, each stage with implications for recruitment procedures, but the authors conceded that more theoretical and empirical research were required to test its usefulness [19]. Little is known empirically about how preferences are expressed by patients during RCT appointments and whether these can be addressed to improve the levels of recruitment.

We investigated how treatment preferences were expressed and discussed with recruitment staff during routinely audiotape-recorded recruitment appointments in a multicenter RCT of treatments for localized prostate cancer (the ProtecT [Prostate cancer testing and Treatment] study). These appointments were “real-life” interactions between recruiters and potential RCT participants and enabled a detailed prospective investigation using qualitative research methods of how preferences were initially expressed and justified, how they changed during recruitment discussions, and how they impacted on participation and treatment decisions. Insights from these dynamic interactions provided a framework for investigating the role of treatment preferences in informed consent and RCT recruitment.

## 2 Patients and methods

### 2.1 Study group

In the ProtecT study, overall 2,698 men aged 50–69 years and diagnosed with localized prostate cancer after community-based prostate-specific antigen (PSA) testing attended an appointment with a study nurse to consider recruitment to an RCT comparing radical prostatectomy, radical conformal radiotherapy, and active monitoring of PSA levels (<1% of eligible men did not attend) (for further details, see Ref. [20]). Before the appointment, men were provided with a detailed written patient information sheet containing details about treatments and the need for an RCT. Recruiters were research nurses, predominantly female, with many holding senior positions and having previous experience of research. Nurses were given training and feedback to ensure that they provided accurate and detailed information about the study and treatments and to enable recruitment to be as uniform as possible across the different centers. A checklist was provided to remind them of the essential study information concerning diagnosis, advantages and disadvantages of treatments (including those outside the trial), the need for an RCT, the purpose of randomization, and the right to refuse participation or take time to consider. They were encouraged to elicit and explore potential participants’ preferences before assisting them in reaching an informed decision about participation or treatment [14,20]. If men expressed a clear preference for one of the treatments or were not willing to be randomized, nurses enabled them to select a treatment; if they were sufficiently uncertain and willing to consider all three treatments, they were invited to have their treatment randomly allocated. The ProtecT study was designed as a comprehensive cohort RCT [21]—all those diagnosed with prostate cancer (randomized or not) were followed up in the same way.

### 2.2 Data collection and analysis

Recruitment appointments in the ProtecT study were routinely audiotape recorded for training and monitoring purposes [22]. This enabled a systematic assessment of interactions between participants and recruiters and, in this analysis, particular focus on treatment preferences. All recruitment appointments across all nine clinical centers over a 3-month period (October to December 2005) were included in this qualitative study. Men attended one appointment (with the exception of two men who attended two appointments), lasting between 30 minutes and 2.5 hours, with most lasting an average of 90 minutes. Variation across the sample was assessed in relation to routine study data available on age, marital status, ethnicity, grade of tumor (Gleason score), and center. Sociodemographic data were obtained from questionnaires completed at the time of PSA testing (ie, before diagnosis), and the UK National Statistics Socio-Economic Classification system was used to assign a socioeconomic group on the basis of their occupational title and responsibilities [23]. The relationship between preference categories and study center, sociodemographic data, and cancer staging was assessed using chi-square, Fischer exact test, and *t*-test techniques in Stata v11 (StataCorp LP, College Station, TX, USA).

N.M. initially listened to all recruitment appointments to identify the initial expression and justification of treatment preferences, which were then compared with the outcome of the appointment. However, treatment preferences were not expressed as simply as the systematic reviews had suggested [7,9]. Although most men did express a view about treatments early in appointments, most were hesitant about their views and some did not express a preference at all. The definition of a “treatment preference” was problematic because there was variation in the way it was expressed between participants and over time. A more detailed formal qualitative analysis was then undertaken by N.M. to investigate how the treatment views or preferences were expressed (in favor or against a treatment), when they were uttered, how strongly they were held and justified, and what happened to these views/preferences during discussion with the recruiter. The relationships between the expressed views/preferences and the decision to be randomized and the treatment the participant finally agreed to were also explored. Analysis was an iterative process—a repeated cycle of coding and interpretation. Verbatim transcripts of recruitment appointments were prepared, and data items were systematically assigned codes using the qualitative data organization package Atlas.ti V5 (Scientific Software Development GmbH, Berlin, Germany). At this point, a second experienced social scientist (J.W.) analyzed 10 random recruitment appointments (approximately 10% of the data) independently to compare coding and enhance its reliability. Areas of disagreement (few were major) were discussed and resolved, and the coding framework was refined and reapplied to the data. Similar codes were grouped to produce categories or themes using content analysis techniques [24] and methods of constant comparison [25]. Instances where there was apparent contradiction between the initial treatment preference and the outcome of the appointment were identified separately as potential negative cases [26] and reanalyzed in detail using audio and transcribed data to gain a deeper understanding of the data and increase the trustworthiness of the findings. N.M. and J.L.D. met regularly to review findings in detail and discuss theoretical development. The findings are displayed as flow charts and in analytic accounts below. Further quotations to support data interpretation are in Appendix on the Web.

## 3 Results

During the specified period, 108 recruitment appointments were conducted and 93 were recorded successfully. The appointments were broadly representative of the ProtecT study: they were conducted in all study centers and included participants across the age range, in working class and managerial/professional positions, with the commonest grade of PSA-detected localized prostate cancer (Gleason score 6), and married or living as married (Table 1). In terms of the outcome of recruitment, they were also broadly representative as 69 (74%) agreed to be randomized (60 of whom accepted the allocated treatment) and 24 (26%) selected their treatment [20]. The findings are presented below in three sections. First, issues related to the difficulty of defining simple treatment preferences are presented. Second, the dynamic nature of the treatment views and preferences is shown, including how they changed over time and in response to interactions with recruiters. Finally, treatment views and preferences are considered in relation to the outcome of the appointments—whether patients were recruited to the RCT or chose a treatment.

### 3.1 Treatment views and preferences: simple categories or a complex continuum?

Most participants (64 of 93 [69%]) expressed views about treatments early in the appointments, based on information gleaned from the study patient information sheet, lay sources, general practitioners, or media/Internet (Appendix w1):

ID 69: “I thought because you heal better as you're younger and fitter … if you're going to have anything major you're best doing it while your body’s fit enough … might as well get it all done now [surgery] while I’m fit, because you never know with your health do you.” (Study center F)

Sometimes men expressed a view with clarity (Appendix w2):

ID 80: “Surgery rids the prostate and therefore rids the cancer. I would be worried about it spreading if I had active monitoring.” (Study center I)

But other men expressed views more hesitantly (Appendix w3):

ID 63: “If I did have anything done, I would prefer the surgery …. I don’t know that much about it but I think if you have surgery umm probably they could remove something umm (pause) and I, I would sooner have that then the uhh radiotherapy or the uhh monitoring because this is just my personal view.” (Study center D)

Some expressed views that were not concordant with scientific evidence:

ID 35: “[Having surgery] puts cancer some place else … [Radiotherapy] makes all your hair fall out.” (Study center I)

Expressed preferences thus varied from relatively unformed views about treatments to clear requests for a treatment. Each of the quotations above could be said to contain a treatment “preference”—defined as an opinion about a potential treatment option. However, many of the views contained caveats or were limited by the lack of clarity from the source of information, and so it would be difficult to claim that any of these treatment “preferences” were fully informed. Indeed, several men were explicit that they were expressing a view rather than a preference (see also ID 63 above):

ID 26: “Active monitoring sounds to me like the right thing to do. That’s what I feel at the moment. I’m sure I could be talked out of it.” (Study center G)

If recruiters had simply accepted these views as “preferences” without further exploration, this would likely have resulted in these men choosing a treatment. However, appointments in this study were conducted within an RCT in which recruiters were trained to listen to and acknowledge these opinions, explain the reasons for the RCT, and present detailed information about each treatment to ensure that a fully informed treatment or RCT participation decision was reached [20]. The recordings of the appointments made it possible to investigate prospectively what happened to treatment views expressed early on in response to information provided by recruiters.

### 3.2 The development of “views” into treatment preferences or uncertainty

**Fig. 1:**
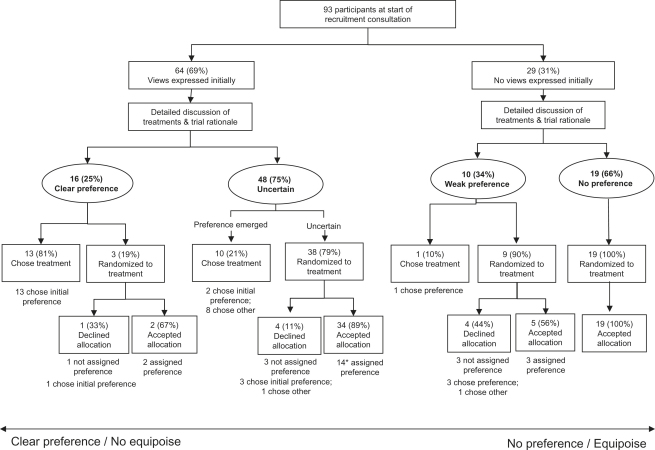
The development or dissipation of participants’ treatment preferences during (and in some cases after) the recruitment appointments. ∗ indicates that one man did not state what treatment his preference referred to.

As appointments proceeded, initial treatment views and preferences were explored by participants and recruiters in the context of the study information. Two clear groups of participants from across all study centers then emerged—those who sustained or developed a clear treatment preference and those who remained or became uncertain (Fig. 1). There were no differences in terms of sociodemographic characteristics or cancer staging between the two groups with the exception of socioeconomic status: men who sustained or developed a clear treatment preference were more likely to be in managerial/professional occupational positions than those who remained or became uncertain (11 of 15 [73%] vs. 17 of 47 [36%]; *P* = 0.02).

#### 3.2.1 Participants who sustained or developed a clear treatment preference

There were 16 participants (25% of those expressing views initially) whose views became focused into a clear preference for one treatment:

ID 01: “In my head you've now firmed everything up … by electing to stay in the study I’m not in control. I've always been in control of everything and that is why I wish to opt out of the study and go for regular monitoring … I don’t think at this stage I need [further information] cos I have made up my mind. I’m quite happy that it’s monitoring.” (Study center A; chose active monitoring in consultation)

**Fig. 2:**
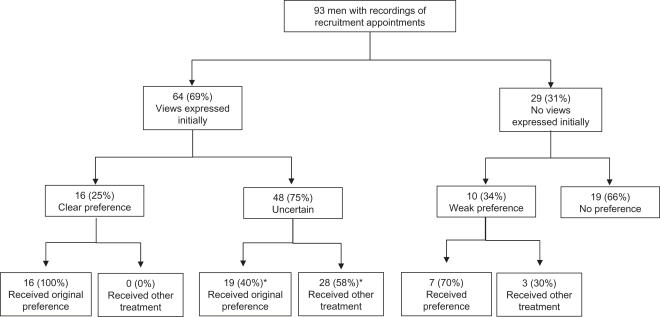
Participants’ treatment preferences in relation to outcome of trial recruitment. ∗ indicates that one man did not state what treatment his preference referred to.

All 16 participants who expressed such a clear preference obtained the treatment they wanted (Fig. 2). As can be seen in Fig. 1, 13 patients chose their treatment outside the RCT and 3 were recruited. These three should probably not have been randomized. Detailed examination of their appointments revealed that they expressed their treatment preference clearly but also insisted that they wanted to remain in the study and contribute to it fully by being randomized. Two were then allocated, by chance, to their preferred treatment and accepted it; the other declined the “wrong” allocation and chose his preferred treatment. Ultimately, all these participants held preferences strong enough to ensure that they received their preferred treatment.

#### 3.2.2 Participants who became uncertain

Most (48 [75%]) of those who had initially expressed views about treatments became uncertain as recruiters provided them with more information (Fig. 1). This increasing uncertainty meant that participation in the RCT became more acceptable [Appendix w4]:

ID 52: “When I came in I thought I'll get surgery and have done with it … but I am listening to you and now I've swung towards the radiotherapy … The monitoring would be nice, um, but I just need something to be done … and I’m not happy to go through an operation which, if the radiotherapy works, I wouldn’t have had to have had … Well you've given me another alternative to how I was thinking.”

RECRUITER: “So you're feeling more open to the radiotherapy?”

ID 52: “Yes yes.”

RECRUITER: “And a little bit open to the active monitoring?”

ID 52: “Yes … well it’s reassured me ….”

RECRUITER: “And a bit open to the surgery ….”

ID 52: “That’s right.” (Study center E; randomized to radiotherapy, accepted allocation in consultation)

As can be seen in the quotation above and Fig. 2, many of these participants accepted treatment that differed from their first expressed view/preference. Of the 48 who had expressed a view and then became uncertain, 38 (79%) agreed to be randomized (Fig. 1), and of these, 34 accepted the allocation. Scrutiny of the appointments of the four participants who refused the random allocation revealed evidence of uncertainty in their appointments and willingness to consider all three treatments at the point of randomization, but when the allocation was given to them, they requested time to reconsider. Three later rejected the allocation and opted for their initially expressed view; the other chose a treatment different from their original view. The process of being allocated to a treatment appeared to help focus their minds on which treatment they could really accept.

The remaining 10 men who became uncertain as the appointment progressed decided to choose a treatment (Fig. 1). Like other uncertain participants, their initial views evolved after provision of further information by recruiters. These participants were unwilling to accept randomization as a method to determine their treatment or expressed serious reservations about one or more of the treatments, meaning randomization was not a suitable option (Appendix w5). Eight of these 10 chose a treatment different from their originally expressed view, evidence that treatment choice was revised in response to information provision and that the ultimate choice was, therefore, more informed.

### 3.3 Relationships between treatment views/preferences and recruitment outcome

Some relationships between early expressed treatment views and the outcome of the appointments were relatively straightforward, particularly at the extremes illustrated in Fig. 1. There were, for example, 13 participants whose initial view was sustained and they chose their preferred treatment; similarly, there were 19 who did not express a view about treatments at all and then agreed to be randomized and accepted the allocation (Fig. 1). In the latter group, scrutiny of their appointments confirmed that they were given detailed information by recruiters from the different study centers and had had opportunities to ask questions, but they appeared content with recruitment to the RCT and did not express a treatment preference. These men were less likely to hold managerial/professional occupational positions than the remaining men who expressed some kind of preference (3 of 19 [16%] vs. 30 of 71 [42%]; *P* = 0.04), but they did not differ with respect to other sociodemographic characteristics or cancer staging.

In most cases, the relationship between the early expressed treatment view and the ultimate outcome of the appointment was not easily predictable or simple (Fig. 2). Among the 48 who became uncertain during the appointments, for example, 19 received their originally expressed preference, but 28 accepted a different treatment (Fig. 2). Randomization appeared to help when an early preference was replaced by uncertainty:

RECRUITER: “If you've got an absolute fixed sense of what you want to do then we won’t bother with randomisation ….”

ID 06: “I think I'll, I would like to have it eradicated and go for radiotherapy. That’s just my feelings at the moment … with the monitoring it’s still there, I’d like to get rid of it … [Discussion about active monitoring] …. The good thing I suppose with active monitoring you know one or two years down the line it’s still the same, so it can be the same in the next couple of years ….”

RECRUITER: “…. You say that you're sort of focused on the radiotherapy, but it sounds to me that, I can see in some ways that each of these [treatments] appeal on different levels.”

ID 06: “That’s right yeah.”

RECRUITER: “So you would be able to think about all of these?”

ID 06: “Yeah that’s right.”

RECRUITER: “So therefore perhaps let’s see what comes up with the randomisation and you can then make a decision, accept or not?”

ID 06: “Yeah, yeah.” (Study center H; randomized to surgery, accepted after consultation)

In the above excerpt, the recruiter could have considered that the initially expressed “preference” for radiotherapy should have precluded participation in the RCT. However, further discussion discovered uncertainty and meant that recruitment became a reasonable option. Randomization then helped this man (and others) to focus their views on the acceptability of a particular treatment.

There was a further group of 10 participants across five different study centers who did not express a view originally and then became rather indecisive, expressing a mix of uncertainty and rather weak noncommittal views (Fig. 1). Most (nine) agreed to randomization, but they split almost equally in terms of whether they accepted/rejected the allocation and whether they received the treatment they seemed to prefer (Fig. 1). Detailed scrutiny of these appointments suggested that these participants had considerable difficulty deciding on participation or treatment:

ID 81: “I would prefer to be monitored but the other two [treatments] don’t frighten me in any way.”

RECRUITER: “No, so you've got no strong objections to any of them?”

ID 81: “No, no strong … no, well the treatments don’t bother me, it’s all this time off work.”

[Discussion about time off work]

RECRUITER: “The next step is entirely up to you what to do.”

ID 81: “Hmm, see then [do the randomization].”

(Study center A; randomized to radiotherapy, declined, chose active monitoring after consultation)

In this example, it might be considered that the participant should not have been randomized. However, the expression of the “preference” is not much different from the previous example in which the allocation was accepted. In the appointment, the recruiter has to make a judgment without the benefit of this detailed examination and hindsight. Moreover, these participants seemed to need randomization and then time after the appointment to consider whether they could accept the allocated treatment.

## 4 Discussion

This study has shown that treatment preferences expressed by potential RCT participants vary along a continuum from hesitant opinions to well-formed intentions to receive a particular treatment and that in many cases, these “preferences” can change after detailed discussion of treatments and trial rationale with recruitment staff. It seems likely from systematic reviews [5,7,9] and low rates of recruitment of eligible participants into RCTs [1] that many of these initial treatment views have been taken on face value to be “simple treatment preferences” and led directly to treatment. However, the prospective nature of this research has shown that these views were far from simple or static and that when these views were elicited and discussed, there was only a weak relationship between participants’ initial views and the treatments finally received. Only a small minority of participants did not express treatment views, seemingly content to accept recruitment to the RCT. The vast majority did express their views and discussed them willingly with recruiters trained to explore their preferences in the context of available evidence. During these interactions, the quarter with strong views justified them and went on to receive their preferred treatment. The other three-quarters became uncertain, and after further discussions with recruiters, most agreed to be recruited to the RCT, often eventually accepting a treatment different from their originally expressed “preference.” The process of preference exploration, and in some cases randomization itself, appeared to help to formulate informed decisions.

These findings raise a number of issues about the interpretation of the current literature concerned with treatment preferences. Many of these studies, including several significant systematic reviews [5,7,9], failed even to provide a definition of preferences because they were considered simple in conceptual and measurement terms. Where a definition was provided, such as “a greater liking for one alternative over another,” [27] this failed to encapsulate the range in strength and complexity, and dynamism, of treatment preferences demonstrated in this study. This lack of adequate definition of preferences may explain the lack of progress so far noted in this field [8]. There is now greater credence to the small number of studies that have critiqued this research and attempted to investigate the complexity of treatment preferences [11–13,19].

Previous studies have shown that treatment preferences have an impact on RCT recruitment [5–7]. This study extends and clarifies this by showing that it is not just the existence of a preference that is important but the type and strength of it and how it is then dealt with by recruitment staff. In a systematic review of musculoskeletal RCTs, 57% of participants were considered to have expressed a treatment preference [9]. This is similar to the 69% expressing initial treatment views in this study. However, in this study, only one-quarter of those with these early “views” clearly upheld them and received their preferred treatment. If such initial, rather ephemeral “likes/dislikes” early in the process of recruitment are accepted to be treatment preferences, then large numbers of potential participants will be excluded from RCT recruitment—effectively half the participants in this study. As many trials fail to reach their original recruitment targets [1], application of the methods of preference exploration used in this study could potentially considerably increase recruitment.

There is a balance to be struck in exploring treatment preferences. If strongly held, informed preferences are elicited from potential recruits, modern ethical standards [4] mean that they should not be asked to consent to randomization but should receive their treatment of choice. Exploring the origin and basis of such preferences has been suggested to be potentially coercive [28]. There is considerable evidence in this study that participants were not coerced. For example, those whose views were strengthened during the interactions all obtained their preferred treatment, even when randomly allocated to a treatment they did not favor. Likewise, for those who became uncertain after expressing an initial view or were uncertain at the start, the process of recruitment and/or randomization helped to focus their views into decisions. Exploration of initial views about treatments in this study showed that some were based on a misunderstanding of scientific evidence. Uncritical acceptance by recruiters of initial views essentially excludes potential participants from provision of full and accurate treatment information—and this, in turn, could be seen to be unethical [14].

In the current literature, there is also interest in the effect that patients’ treatment preferences can have on trial outcome based on the theory that those with preferences who consent to randomization may comply better and have better outcomes if they are allocated to their preferred option [29] or suffer “resentful demoralization” with lower motivation and poorer outcome if allocated to another treatment [30]. The “fully randomized preference trial,” in which participants’ preferences recorded before randomization are taken into account in the analysis, attempts to measure such effects (see, eg, Refs. [31–33]). However, any effect on outcome from such studies will depend on what sort of “preferences” are measured, and as potential RCT participants expressing a clear treatment wish should not be randomized, this exposes a paradox. If preferences are so weak that it is ethical for patients to be randomized, it becomes difficult to imagine how such ephemeral views could have an impact on outcomes such as symptoms or quality of life. Research on treatment preferences in the context of outcome will need to be reconceptualized in light of these study findings.

This study has some limitations. It was set within a single RCT where training had been provided to encourage discussion of treatment preferences. This is a limitation in terms of generalizability, but it enabled the key issues of the definition of “preferences” and their complexity and dynamism to be uncovered, and these aspects are likely to be of relevance to other RCTs. The study was unusual in analyzing routinely audiotape-recorded appointments rather than participant and recruiter interviews. This limited the study as it was not possible to pursue more about participants’ preferences and reflections on their definition in interviews, but it did avoid reliance on post facto rationalizations that have seriously limited interview-based studies in this area [34]. The use of routinely recorded appointments also enabled a systematic and prospective qualitative analysis of a relatively large number of recruitment appointments across nine centers and so was able to provide insights, uniquely, from real-life appointments observed as they happened.

Several methods were used to enhance the reliability and trustworthiness of the data, including the iterative process of going back and forth between coding and interpretation as analysis proceeded, the use of the computer package Atlas.ti to help elicit and categorize findings in an exploratory and systematic way, the active search for negative cases, analyzing both audio and transcribed verbatim data for each case, regular discussions of coding and emerging findings within the research team, and extensive presentation of raw data from different informants so readers can judge the interpretation of data. The consistency of the findings across centers suggests that the styles of individual recruiters were not particularly influential, but in the study, it must be remembered that recruiters were trained together. The finding that men in managerial employment were more likely than those with other occupations to have a clear treatment preference was consistent with other research [7,35].

Findings from this study may be applicable more widely than recruitment to RCTs. The process of shared treatment decision making in routine clinical practice involves a balanced consideration of patients’ treatment preferences and information/evidence provided by clinicians. In areas of clinical practice, perhaps particularly where there is uncertainty, awareness of the continuum on which patients’ preferences might lie might enable clinicians to elicit and better understand patients’ views so they can inform them of treatment options in a more targeted way.

Future research, directed first to RCT recruitment, needs to focus on developing interventions to improve RCT recruitment and informed consent, within the context of dynamic treatment preferences and their role in treatment decision making. It is possible to develop training sessions to encourage recruiters to explore patients’ treatment preferences so information can be tailored, which may, in turn, result in more cost-effective RCT recruitment and more informed decision making [20]. Findings from this study suggest that the group of patients who may benefit the most from exploration of preferences are those who express a desire for a treatment early in the consultation but reveal hesitancy or uncertainty as detailed treatment and trial rationale information is given. Recent research has revealed effective communication techniques used by trained recruiters, for example, open questions, long pauses, and readily ceding the floor, that have facilitated detailed and systematic exploration of potential trial participants’ concerns and understandings [14]. The key for future research is to develop strategies to support recruiters in carefully eliciting and exploring treatment preferences, building on the techniques described above, so that they can identify the more robust preferences from the ephemeral views and provide targeted information to those who need it most.

## 5 Conclusion

This study has provided detailed empirical evidence to show that potential RCT participants’ treatment preferences range on a continuum from ephemeral likes/dislikes to a determination to receive a particular treatment. Preferences are dynamic and can change after detailed discussion of treatments and trial rationale with the recruitment staff. The impact of potential participants’ treatment preferences on recruitment needs to be reconsidered not as a “barrier” to recruitment but as an integral part of the information exchange necessary for informed decision making about treatments and RCT participation. The evidence presented here suggests that exploring patients’ views and preferences about treatments and targeted information provision could lead to improved levels of informed consent and RCT participation.

## Figures and Tables

**Table 1:** Study sample characteristics (*n* = 93)

Patient characteristics	*n*	%
Age at recruitment appointment (y)		
50–54	5	5
55–59	15	16
60–64	38	41
65–70	35	38
Mean age (SD)	62	4.56

Ethnicity		
White	91	98
Other	2	2

Marital status		
Married/living as married	76	82
Single/widowed/divorced/separated	17	18

Socioeconomic status^a^		
Managerial and professional	33	37
Intermediate	14	16
Working	43	48

Grade of cancer (Gleason score)		
6 (lower risk)	72	77
7/8^b^ (higher risk)	21	23

Study center		
A	9	10
B	9	10
C	7	8
D	6	6
E	17	18
F	16	17
G	9	10
H	11	12
I	9	10

*Abbreviation*: SD, standard deviation.

a Based on the UK National Statistics Socio-Economic Classification (three-class version) [23]—three missing observations.

b Only two participants had grade 8.
