# Supplementary material for: Exploring treatment preferences facilitated recruitment to randomized controlled trials
Source: J Clin Epidemiol. 2011 Oct;64(10):1127–36. doi: 10.1016/j.jclinepi.2010.12.017 (PMC3167372; doi:10.1016/j.jclinepi.2010.12.017)
Supplement: Appendix [file mmc1.pdf]

Appendix 1 (web only): Further quotations to support data interpretation

[W1]: *Id 59: "I had a chat with [GP]… first of all I told him what I was thinking [to opt for active monitoring] and umm he seemed quite happy with that. He said you've not got a high risk thing, or high grade …. perhaps if you feel that way, go that way."* (Study centre E)

[W2]: *Id 66: "[My cancer is] so low grade that I would be a fool to have an operation or radiotherapy with the side effects."* (Study centre D)

[W3]: *Id 86: "My inclination at the moment quite honestly is I would rather be monitored to see whether anything happens. Why would I opt for these very drastic moves when I thought up until Wednesday I was relatively healthy and had none of the other side effects? But that's my sort of gut reaction to these things."* (Study centre G)

[W4]: *Id 22: "I initially thought of monitoring, that was my immediate reaction. In other words, just go and get a wee jab and then react to that... Then I thought that um the option I was going for was the surgery – um mainly because the seven and a half weeks duration [of radiotherapy]"*
RECRUITER: "So what are you thinking? Would you consider being randomised within the study itself?"
Id 22: "Oh well as part of the study I'd have to say yes, because I find the study's been a brilliant thing..."
RECRUITER: "If you were randomised and it was radiotherapy then you'd choose?"
ID 22: "I would go with it [the randomised treatment]."

(Study centre B; randomised to radiotherapy, accepted allocation in consultation)

[W5]: *Id 69: "The initial reaction I had as soon as he said [cancer] I thought well I want [prostate gland] out….. quicker out the better…..*

[Recruiter describes all treatments]

The only thing that puts me off um the radiation really is that it's something that mentally I don't like. I'm the sort to take it out …. I think that I'm still tending to lean towards uh surgery...

[Recruiter discusses active monitoring]

Now that I know [prostate cancer] is there, in my own mind I want to get rid of it as quickly as I can. One thing that has crossed my mind uh is that obviously I'd thought that radical surgery and just getting it out might be possibly the quickest and best route but I'm just wondering if the radiography might be, looks lesser side effects as I see it, you know the blood clots and all of that…. Is it possible for me to put myself forward for that [radiotherapy]?"

RECRUITER: "There's no way you'd consider active monitoring at all?"

Id 69: "No I wouldn't be able to absorb that in my mind….I don't think I could sit waiting, not knowing that it's there."

(Study centre F; chose radiotherapy in consultation)
